# SALO, a novel classical pathway complement inhibitor from saliva of the sand fly *Lutzomyia longipalpis*

**DOI:** 10.1038/srep19300

**Published:** 2016-01-13

**Authors:** Viviana P. Ferreira, Vladimir Fazito Vale, Michael K. Pangburn, Maha Abdeladhim, Antonio Ferreira Mendes-Sousa, Iliano V. Coutinho-Abreu, Manoochehr Rasouli, Elizabeth A. Brandt, Claudio Meneses, Kolyvan Ferreira Lima, Ricardo Nascimento Araújo, Marcos Horácio Pereira, Michalis Kotsyfakis, Fabiano Oliveira, Shaden Kamhawi, Jose M. C. Ribeiro, Nelder F. Gontijo, Nicolas Collin, Jesus G. Valenzuela

**Affiliations:** 1 Department of Medical Microbiology and Immunology, University of Toledo College of Medicine and Life Sciences, Belo Horizonte, MG, Brasil; 2 Laboratório de Fisiologia de Insetos Hematófagos, Departamento de Parasitologia, Universidade Federal de Minas Gerais, Belo Horizonte, MG, Brasil; 3 The University of Texas Health Science Center at Tyler, Tyler, TX; 4 Vector Molecular Biology Section, LMVR, National Institute of Allergy and Infectious Diseases, NIH, Rockville, MD; 5 Biology Center of the Czech Academy of Sciences, Budweis,CZ-370 05, Czech Republic; 6 Vector Biology Section, LMVR, National Institute of Allergy and Infectious Diseases, NIH, Rockville, MD; 7 Vaccine Formulation Laboratory, Department of Biochemistry, University of Lausanne, Switzerland

## Abstract

Blood-feeding insects inject potent salivary components including complement inhibitors into their host’s skin to acquire a blood meal. Sand fly saliva was shown to inhibit the classical pathway of complement; however, the molecular identity of the inhibitor remains unknown. Here, we identified SALO as the classical pathway complement inhibitor. SALO, an 11 kDa protein, has no homology to proteins of any other organism apart from New World sand flies. rSALO anti-complement activity has the same chromatographic properties as the *Lu. longipalpis* salivary gland homogenate (SGH)counterparts and anti-rSALO antibodies blocked the classical pathway complement activity of rSALO and SGH. Both rSALO and SGH inhibited C4b deposition and cleavage of C4. rSALO, however, did not inhibit the protease activity of C1s nor the enzymatic activity of factor Xa, uPA, thrombin, kallikrein, trypsin and plasmin. Importantly, rSALO did not inhibit the alternative or the lectin pathway of complement. In conclusion our data shows that SALO is a specific classical pathway complement inhibitor present in the saliva of *Lu. longipalpis*. Importantly, due to its small size and specificity, SALO may offer a therapeutic alternative for complement classical pathway-mediated pathogenic effects in human diseases.

Saliva of blood feeding insects is a rich source of pharmacologically active components. These biologically active molecules include vasodilators, inhibitors of platelet aggregation, and inhibitors of the blood coagulation cascade that help the insect to get a blood meal by disarming the host hemostatic system^1^,^2^. Other molecules include novel serotonin and leukotriene binding proteins^3^,^4^. It is not surprising that among this arsenal of molecules, saliva of blood feeding arthropods have molecules that inhibit the complement system. In fact, anti-complement activity has been reported in saliva^5^,^6^ and intestine^7^ of blood feeding arthropods, and some were shown to protect insect intestinal cells against complement^7^,^8^.

The complement system, a component of the innate immune system, is one of the first lines of defenses against pathogens. It has important housekeeping functions and provides support to the adaptive arm of the immune system^9^,^10^. The complement system has three main pathways, the classical, the alternative and the lectin pathways. Complement activation results in direct killing of target cells marked with ligands (C3b, iC3b, C3d, C4b) for innate cellular receptors (CR1/CD35, CR2/CD21, CR3, CR4, CRIg) and the induction of numerous adaptive cellular and humoral responses^11^,^12^. Beyond its direct action against microorganisms, the complement system is involved in other functions, such as removal of immune complexes, clearance of necrotic or apoptotic cells, and contribution to antigen presentation. This last function makes the humoral response much more efficient^13^. Sub-products generated during complement activation (the anaphylatoxins C3a and C5a) are important for phagocyte recruitment to infection sites^11^,^14^ and produce microcirculatory alterations. In particular, these alterations include vasoconstriction, platelet aggregation, and increased vascular permeability^15^. These effects may directly affect the fly’s ability to blood feed.

Activation of the classical complement pathway begins when the C1 complex (composed of C1q, C1r, and C1s proteins) recognizes properly oriented Fc fragments of immunoglobulins bound to antigens as well as other surface patterns including PAMPS or altered self surfaces^16^. Sequential activation of the C1r and C1s serine proteases bound to the collagenous region of C1q follows^17^. Activated C1s then promotes proteolysis and activation of C4 and C2, which is essential for the generation of C3 (C4b2b) and C5 (C4b2b3b) convertases^18^. C5 convertases are then responsible for initiating the subsequent steps required to the formation of the membrane attack complex^18^.

Saliva of *Lu. longipalpis* inhibits the classical pathway of complement^19^. The objective of this work is to identify the salivary protein responsible for the inhibition of the classical pathway of complement in this sand fly species and partially characterize its mechanism of action.

## Results

### SALO (LJM19) is the classical complement inhibitor from the saliva of *Lu. longipalpis*

**Figure 1:**
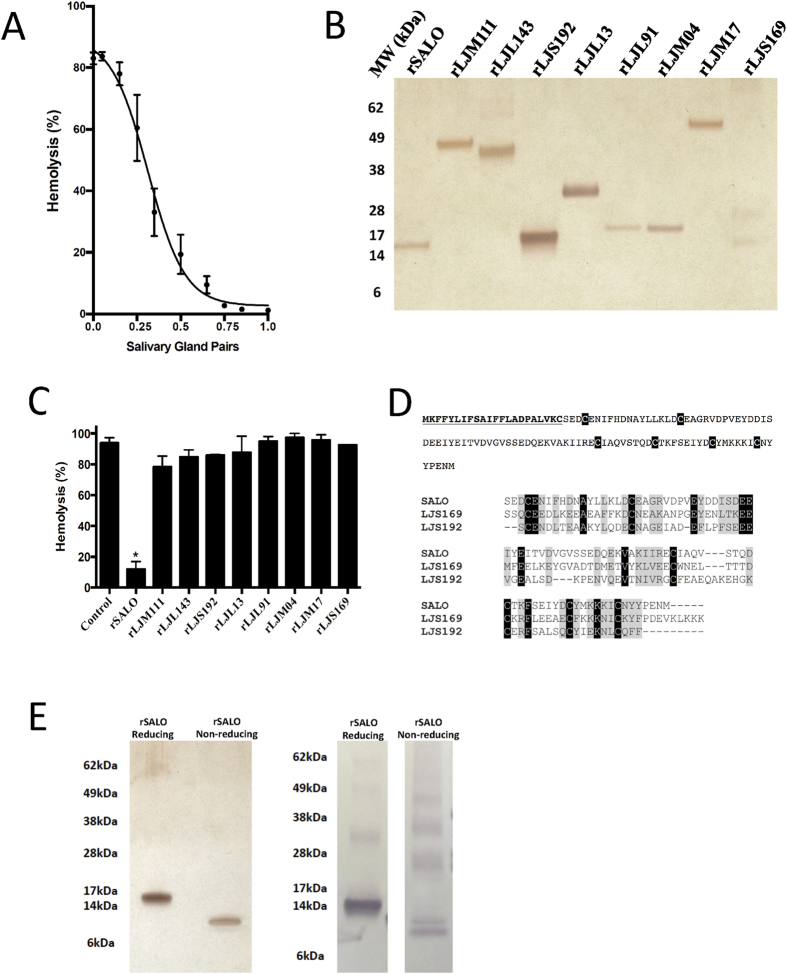
Recombinant SALO (rSALO) inhibits the classical pathway of complement. (**A**) Inhibition of the classical pathway of complement by *Lutzomyia longipalpis* salivary gland homogenate using a hemolytic assay. (**B**) SDS-PAGE loaded with 100ng of distinct *Lu. longipalpis* recombinant salivary proteins expressed on HEK cells (rSALO, rLJM111, rLJL143, rLJS192, rLJL13, rLJL91, LJM04, LJM17, and LJS169) under reducing conditions and stained with silver nitrate. (**C**) Testing various *Lu. longipalpis* recombinant salivary proteins (0.1 μM) on the classical pathway of complement using a hemolytic assay. Erythrocyte lysis was measured at 414 nm. (**D**, top) Primary structure of SALO (AY438271) showing the predicted signal secretory peptide (bolded amino acids) and the cyteines present in the mature protein (black shaded amino acids). (**D**, bottom) Multiple sequence analysis of SALO, LJS169 and LJS192. Black shaded amino acids represent highly conserved amino acids. Light grey shaded amino acids represent similar amino acids. (**E**, left) rSALO run on SDS-PAGE and stained with silver under reducing and non-reducing conditions. (**E**, right) Western blot of rSALO under reducing and non-reducing conditions using anti-rSALO mouse sera. The data for figures **A** and **C** represents the mean plus the standard deviation of three independent experiments.

We first reproduced previous findings and show that the equivalent of one pair (approximately 500 ng of protein) of *Lu. longipalpis* salivary gland homogenate (SGH) is sufficient to inhibit the hemolytic activity of the human classical pathway of complement (Fig. 1A). In order to identify the salivary protein responsible for the observed effect on the classical pathway of complement, we expressed in HEK mammalian cells and purified a panel of recombinant *Lu. longipalpis* salivary proteins that represent the most abundant families of proteins in this sand fly species (rSALO (LJM19), AY43827; rLJM111, DQ192488 ; rLJL143, AY445936.1 ; rLJS192, AY438270.1 ; rLJL13, AF420274 ; rLJL91, AY445934.1; rLJM04, AF132517; rLJM17, AF132518 and rLJS169, AY455912.1) (Fig. 1B). All recombinant salivary proteins were tested in a hemolytic assay for the human classical pathway of complement. Of all the recombinant proteins tested, only rSALO inhibited the classical pathway-mediated lysis (Fig. 1C).

Due to its biological activity, we renamed LJM19 (NCBI accession number AY438271) SALO (Salivary Anti-complement from *Lu. longipalpis*). SALO is a protein of a predicted molecular weight of 10.8 KDa, a theoretical pI of 4.15 with no sequence similarities to other salivary proteins apart from New World sand flies. SALO transcript has a signal secretory peptide (Fig. 1D, top panel) and it contains 6 conserved cysteines (Fig. 1D, top panel). Importantly, the salivary proteins LJS169 and LJS192, which are paralogs of SALO (Fig. 1D, bottom panel), did not show anti-complement activity (Fig. 1C). Recombinant SALO under non-reducing conditions runs at approximately 15 kDa and under non-reducing conditions at around 10 kDa (Fig. 1E, left panel). Antibodies against rSALO under reducing conditions recognize, mostly, a single form of approximately 15 kDa (Fig. 1E, right panel), however, these antibodies recognize different forms of rSALO when this protein is under non-reducing conditions Fig. 1E, right panel); there is a strong recognition of a protein of 10kDa but also other at much higher molecular weight can be observed (Fig. 1E, right panel), suggesting rSALO under non-reducing conditions forms multimers.

### The anti-complement activities of rSALO and *Lu. longipalpis* salivary gland homogenate (SGH) have the same HPLC chromatographic properties

**Figure 2:**
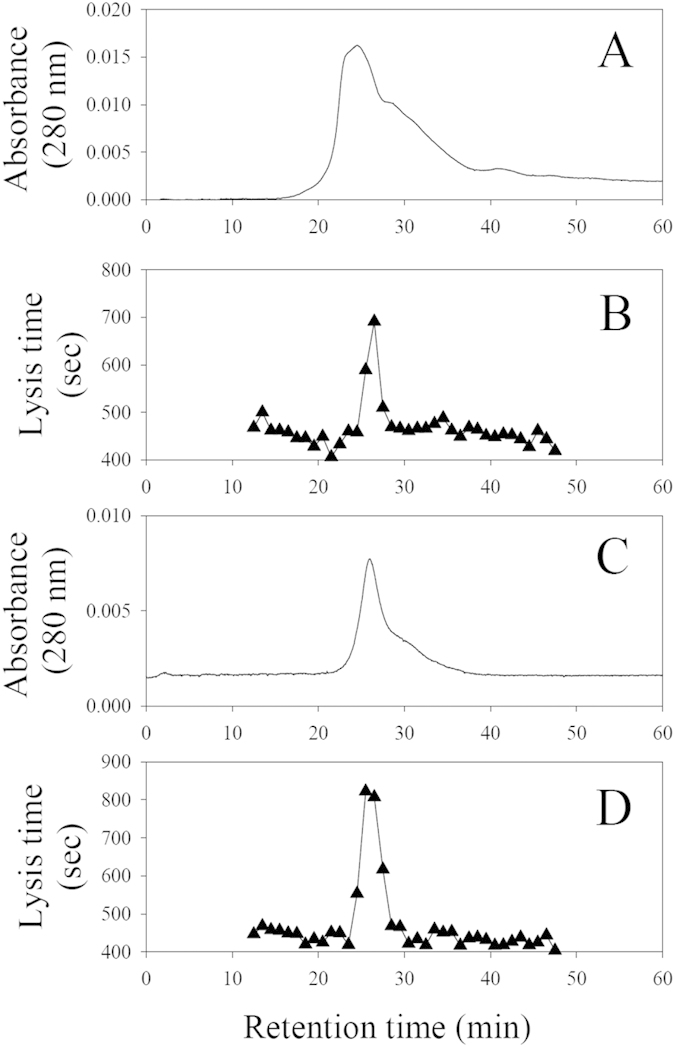
SALO and *Lutzomyia longiplapis* salivary gland homogenate share the same chromatographic features. Molecular sieving chromatography (**A,C**) and screening of classical complement pathway inhibition (**B,D**) by salivary gland homogenate of *Lu. longipalpis* (**A,B**) or by rSALO protein (**C,D**). Lysis time represents the time of erythrocytes lysis induced by complement in a hemolytic assay. The two most active fractions in both chromatograms are the same, namely fractions 26 and 27, producing an average expected MW of 19.6 kDa. Absorbance was measure at 280 nm and erythrocyte lysis at 414nm in a hemolytic assay.

To determine whether rSALO is the protein responsible for the anti-complement activity from the salivary gland homogenate (SGH) of *Lu. longipalpis*, SGH and rSALO were subjected to a molecular sieving HPLC chromatography and all the fractions were collected and tested in a hemolytic assay for the human classical pathway of complement. The HPLC fractions from SGH revealed a sharp peak of inhibition for fractions with a mobility of an average molecular weight of approximately 20 kDa (Fig. 2A,B). The mobility and activity of rSALO (Fig. 2C,D) were observed at a MW similar for the one calculated for the native protein in SGH, suggesting that SALO is the salivary protein on SGH acting on the classical pathway of complement. The chromatographic behavior observed for rSALO and SGH by molecular sieving chromatography shows that the active anti-complement molecule has a higher estimated MW (19.6 kDa) than the predicted molecular weight (11 kDa) of the molecule suggesting the active form of the protein is a dimer or an aggregate that runs at a higher molecular weight. These data is in agreement with the mobility of rSALO observed is SDS-PAGE under non-reducing conditions (Fig. 1E).

### Antibodies against rSALO inhibit and precipitate the anti-complement activity from SGH

**Figure 3:**
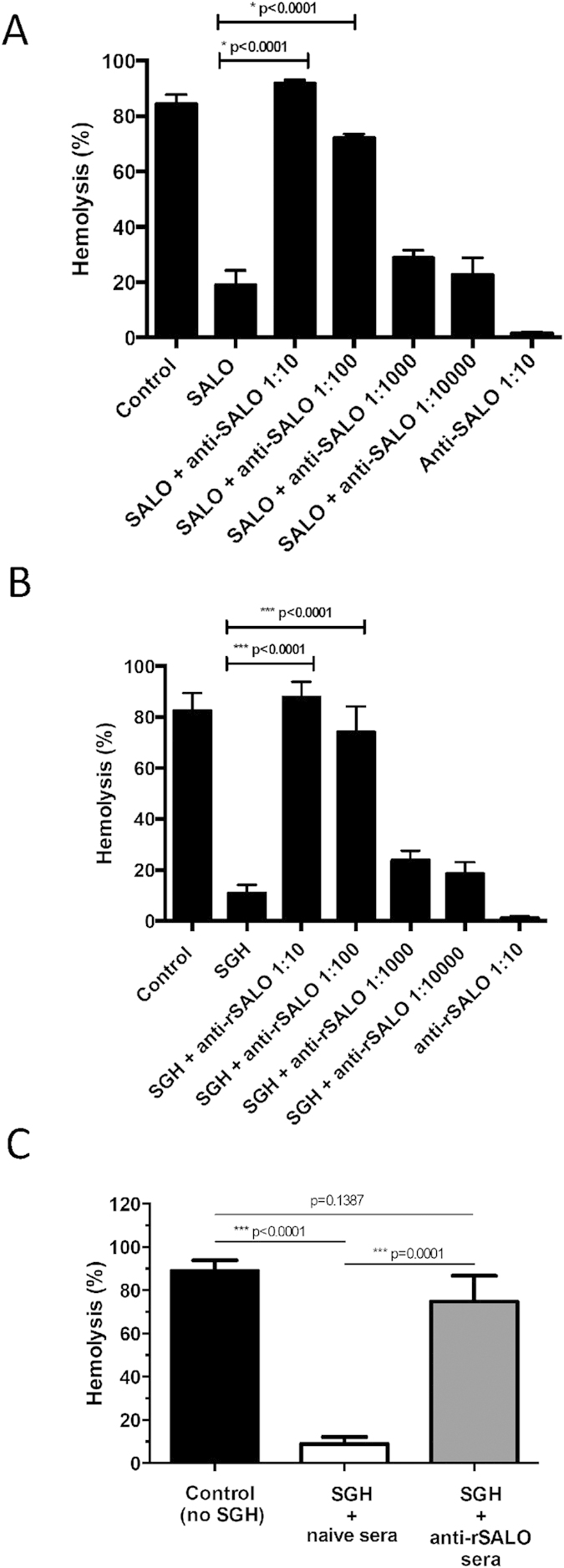
Antibodies against rSALO block anti-complement activity present in the salivary glands of the sand fly *Lutzomyia longipalpis*. Hemolytic assays using rSALO at 0.1 μM final concentration (**A**) or SGH containing 0.5 salivary gland pairs (**B**) in the presence of rSALO antibodies anti-SALO demonstrated inhibition of the anti-complement activity from both rSALO and SGH at 1:10 and 1:100 anti-SALO antibody dilutions (***p < 0,0001, ANOVA and Tukey test). The antiserum alone did not present any hemolytic effect. The data represents the mean ± standard deviation of three independent repetitions. (**C**) Hemolytic assays using SALO-depleted SGH. The data represents the mean ± standard deviation of three independent repetitions. Hemolysis was measured at 414 nm.

Polyclonal antibodies produced against rSALO were incubated with rSALO to test for their effect on its activity. Anti-rSALO antibodies strongly and specifically recognized the native SALO from *Lu. longipalpis* SGH (Supplementary Figure 1) and also the recombinant form of SALO on SDS-PAGE (Fig. 1E). The anti-complement activity of rSALO was inhibited in a dose dependent manner by rSALO anti-sera (Fig. 3A). Similarly when rSALO anti-sera were incubated with SGH, the anti-complement activity was inhibited in a dose dependent manner (Fig. 3B). Furthermore, rSALO antibodies depleted the anti-complement activity of SGH by immuno-precipitation (Fig. 3C) providing further evidence that SALO is the molecule responsible for classical pathway inhibition in *Lu. longipalpis* SGH.

### rSALO and SGH impairs C4 deposition and downstream complement activation

**Figure 4:**
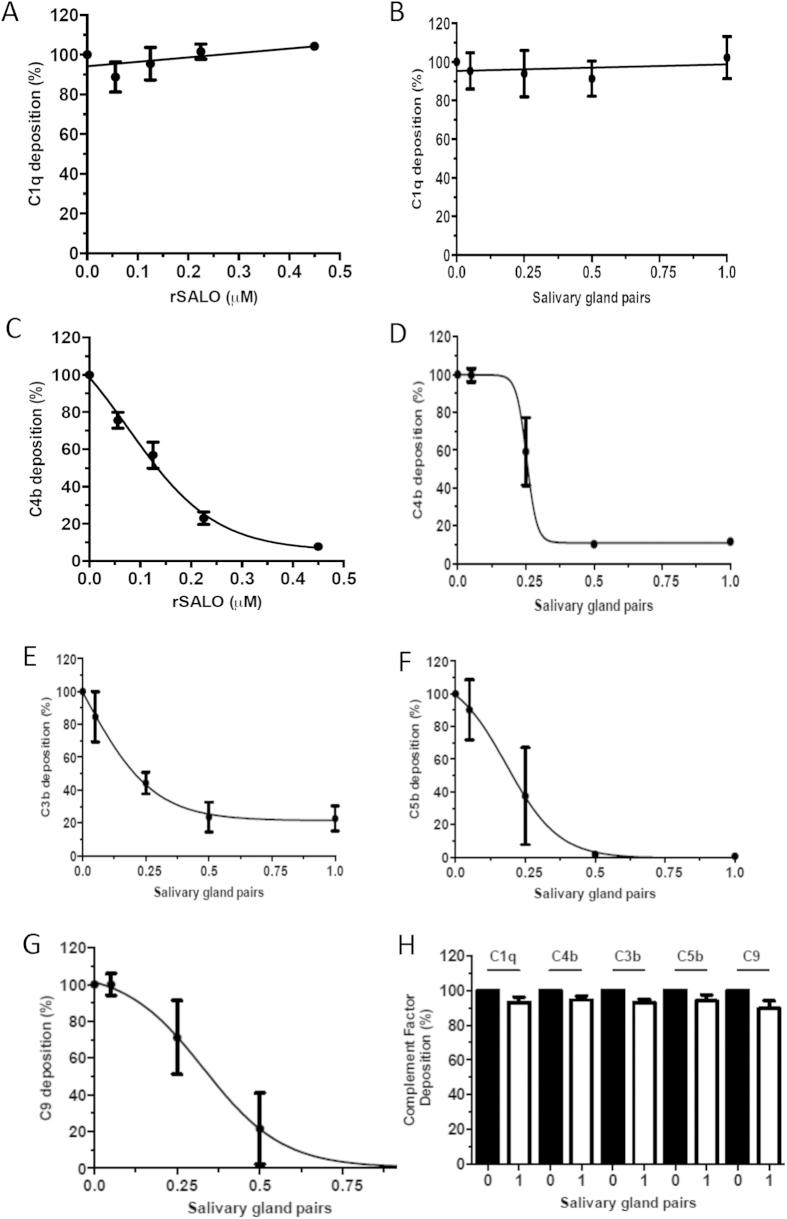
rSALO and SGH effect on the deposition of complement factors activated by the classical pathway. Deposition assays were conducted for C1q (**A,B**), C4b (**C,D**), C3b (**E**), C5b (**F**) and C9 (**G**). Detection of complement factors is not masked by SGH. Incubation with SGH (white bars) or buffer alone (black bars) after complement factors were allowed to deposit does interfere with its detection (**H**). The results represent three independent assays and the bars indicate mean ± standard deviation.

Having demonstrated that SALO is the salivary protein from *Lu. longipalpis* SGH responsible for inhibition of the classical pathway of complement, we tested if rSALO or SGH affect directly the deposition of some of the components on the activation surface of the classical pathway. SGH or rSALO were incubated with 1% normal human serum (NHS) then the mixture was added to IgG-covered microplates and the binding of complement proteins or derived activation fragments was detected using specific antibodies. Both rSALO (Fig. 4A) and SGH (Fig. 4B) did not affect the binding of C1q to IgG; however, both affected the deposition of C4b fragments (Fig. 4C,D respectively). Although, SGH decreased in a dose dependent manner the binding of molecules downstream of C1 activation, including C3b (Fig. 4E), C5b (Fig. 4F) and C9 (Fig. 4G), rSALO did not have any effect on C1q, C3b, C4b, C5b and C9 when these complement proteins were already deposited on the activated surface of the plate (Fig. 4H). This suggests that SALO, present in SGH, impairs the deposition of C4b and consequently impairs the deposition of factors downstream of C4b in the complement cascade.

### rSALO and SGH inhibit C4 cleavage without interfering with C1s proteolytic activity

**Figure 5:**
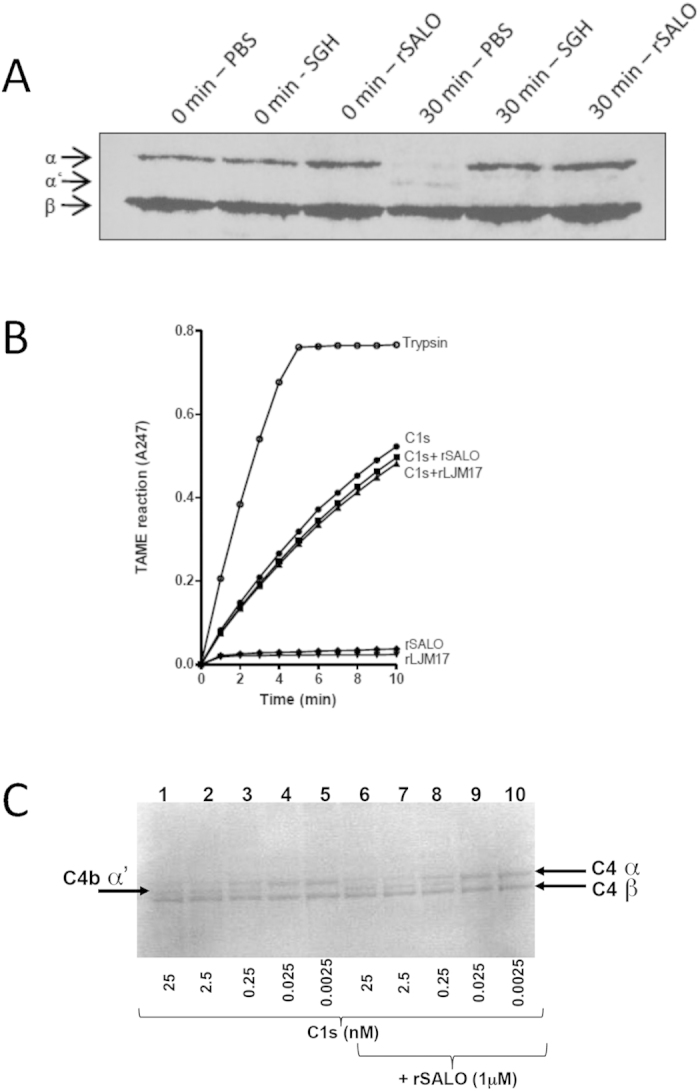
The activity of rSALO and SGH on C4 cleavage and C1s enzymatic activity. (**A**) Hemolytic assays for classical pathway were performed in the presence of SGH (2 salivary gland pairs ≃1 μg of protein), rSALO (0.72 μM) or no inhibitor (PBS control). Supernatants were collected just after addition of sensitized sheep erythrocytes (0 min) or after incubation (30 min), and subjected to 10% SDS – PAGE. Western blot analysis using C4 polyclonal antibody was used to demonstrate C4 cleavage indicated by the presence of C4b α’ chain. (**B**) Pure C1s (0.63 μM) was mixed with rSALO or rLJM17 (0.2 μM) and the substrate TAME added to initiate the enzymatic reaction. C1s activity was assessed by measuring the Absorbance at 247 nm (A_247_) every minute for 10 minutes. Trypsin (0.2 μM) + the substrate TAME was used as a positive control and each recombinant protein in the presence of the substrate TAME, without C1s, was included as negative controls. (**C**) C4 (0.86 μM) and C1s (0.0025–25 nM) were incubated in the presence or absence of rSALO (1 μM). C1s-induced C4b α’chain is present in lanes 1–3 and 6–8 and rSALO does not reduce the presence of these bands.

Classical pathway activation results in C1s–mediated cleavage of C4, generating the larger fragment C4b, which binds covalently to –OH or –NH_2_ groups on carbohydrates and proteins on the cell surfaces on which complement is acting, as well as the generation of the smaller peptide anaphylatoxin C4a^11^. Because C4b deposition was prevented by rSALO, we tested if this was due to rSALO preventing C4 cleavage. Hemolytic assays specific for the human classical pathway of complement, containing all the components of the classical pathway of complement were performed in the absence or presence of rSALO or SGH and the supernatant was collected and analyzed by Western blot using an anti-C4 polyclonal antibody. Activation of the classical pathway led to the formation of the C4b α’ chain band, a product of C4 cleavage by 30 minutes (Fig. 5A, 30 min-PBS); in contrast, the formation of C4b α’ chain band was inhibited when the hemolytic assay was performed in the presence of SGH or rSALO measured also by 30 min (Fig. 5A), indicating that rSALO prevents the cleavage of C4 and the generation of C4b as the C4b α’ chain band was not generated. To test the possibility that impairment of the classical pathway could be due to enzymatic inhibition, assays for C1s serine protease activity in the presence of rSALO were conducted. We used *N*-*p*-tosyl-L-arginine methyl ester hydrochloride (TAME) as a substrate for the C1s enzymatic activity (Fig. 5B). In this assay only the C1s enzyme and the TAME substrate are present in contrast to all components of the complement cascade present in experiments of Fig. 5A. The addition of rSALO or rLJM17, another salivary protein from *Lu. longipalpis* without anti-complement activity (as shown in Fig. 1C), did not decrease the enzymatic activity of C1s (Fig. 5B). Similarly, the same amount of C4b α’ chain was generated when C4 was incubated with C1s in the presence or absence of rSALO (Fig. 5C). Importantly, rSALO does not inhibit other enzymatic activities including thrombin, Factor Xa, Kallikrein, Trypsin, u-PA and plasmin some of which are also related to hemostasis (Table 1) suggesting rSALO inhibitory activity affects only the classical complement cascade and not other important biological hemostatic activities. Taken together, these findings demonstrate that rSALO and SGH containing the native form of rSALO prevent C4 cleavage in whole serum. However, this effect is not due to inhibition of C1s enzymatic activity.

### rSALO does not inhibit other complement pathways

**Figure 6:**
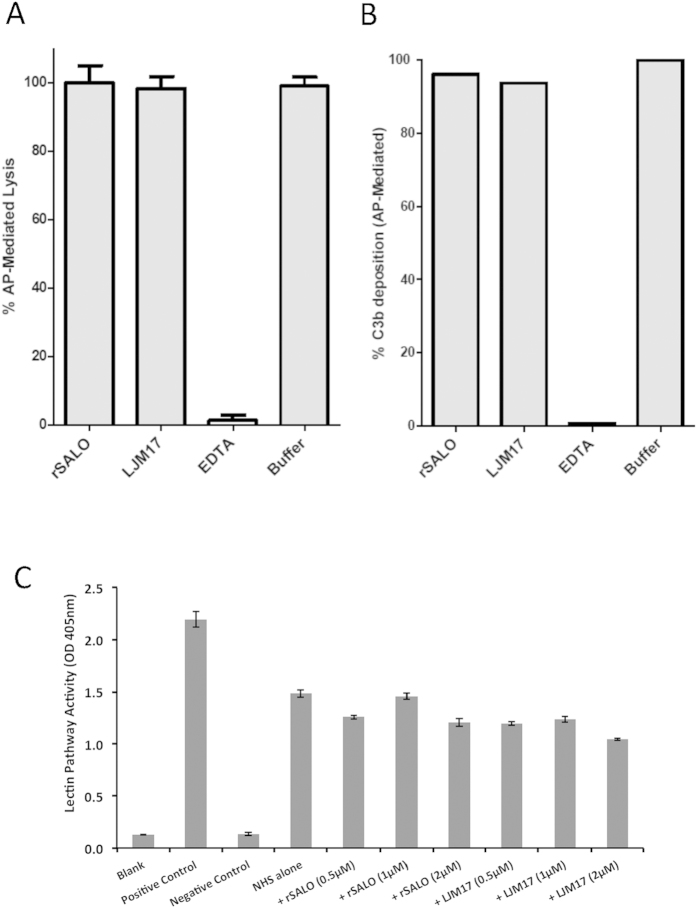
rSALO does not inhibit the alternative or the lectin pathway of complement. (**A**) Evaluation of alternative pathway-mediated rabbit erythrocyte hemolysis in the presence of rSALO. (**B**) Evaluation of alternative pathway-mediated C3b deposition. Each bar represents the mean and standard deviation of triplicate observations. Data shown is a representative experiment of two independent experiments. (**C**) Lectin pathway activity in normal human sera (in the presence or absence of rSALO or LJM17) was assessed using a Wieslab^R^ kit. Positive control sera and negative control sera are provided in the kit. Blank is the dilution buffer alone. Results are representative of 3 independent experiments.

*Lu. longipalpis* SGH was previously shown to inhibit the classical and the alternative pathway of complement^19^. We tested if rSALO, the inhibitor of the classical pathway of complement, could also inhibit the alternative pathway. Hemolytic assays for the human alternative pathway of complement were performed in the absence or presence of rSALO and rLJM17. rSALO did not inhibit the hemolysis caused by the alternative pathway of complement (Fig. 6A), did not inhibit C3b deposition mediated by the alternative pathway (Fig. 6B) and did not inhibit the lectin pathway initiated by MBL when tested at up to 2 μM (Fig. 6C), which is ~10 times higher than the IC_50_ required for classical pathway inhibition (Fig. 6C). Therefore, the data indicate rSALO specifically inhibits only the classical pathway of complement.

## Discussion

Saliva of sand flies and other blood-feeding arthropods contains a variety of potent biologically active molecules, including vasodilators^20^, anti-coagulants^21^ and inhibitors of platelet aggregation^22^ among other biological activities^23^. These salivary proteins block the hemostatic system of the host and allow blood-feeders to get a blood meal. Here we identified SALO, for the first time, as (a) the specific salivary protein of the sand fly vector *Lu. longipalpis* that is responsible for the inhibition of the classical pathway of complement in saliva, (b) capable of inhibiting C4b deposition via the classical pathway, but (c) incapable of inhibiting the alternative and lectin pathways of complement.

Our data indicates that SALO is the protein responsible for classical pathway inhibition as rSALO and *Lu. longipalpis* SGH anti-complement activities have the same chromatographic properties (Fig. 2) both running at a higher than expected molecular weight. This together with the finding that SALO forms oligomers at non-reducing conditions (Fig. 1E) suggests that the multimeric form of SALO may contribute to its anti-complement activity. This point warrants further investigation. Importantly, anti-rSALO antibodies completely blocked and immuno-precipitated the anti-complement activity present in *Lu. longipalpis* SGH (Fig. 3).

SALO appears to be unique to saliva of sand flies belonging to the genus *Lutzomyia* as homologues have been only found in *Lu. ayacuchensis*^24^ and *Lu. intermedia*^25^ sand flies, but not from saliva of sand flies in the genus *Phlebotomus*^26^ or in other organisms. Importantly, the two paralogs present in the saliva of *Lu. longipalpis*, LJS169 and LJS192, did not have anti-complement activity (Fig. 1C, Supplementary Figure 1). This, together with the ability of anti-SALO antibodies to completely inhibit anti-classical pathway in saliva (Fig. 3), indicate that SALO is the only salivary protein from *Lu. longipalpis* SGH that blocks the classical pathway of complement.

This work also “de-orphanizes” the biological activity of a salivary vaccine candidate known to protect rodents against visceral and cutaneous leishmaniasis. SALO was previously known as LJM19, a salivary protein with unknown function^27^. Immunization of hamsters with DNA plasmids coding for the LJM19 protein (now SALO) was previously shown to protect hamsters against the fatality of *Leishmania infantum* infection^28^ and more recently against *Le. braziliensis* infection^29^.

rSALO and *Lu. longipalpis* SGH (containing native SALO) inhibited specifically the classical pathway of complement. Our data indicate that SALO is acting early on in the cascade since SALO and SGH inhibited the deposition of C4b, C3b, C5b and C9, but not the binding of C1q to IgG (Fig. 4). Furthermore, our data also suggest that SALO is not an inhibitor of serine proteases since it did not inhibit the enzymatic activity of C1s nor other important proteases of the hemostatic system including thrombin, plasmin and uPA (Fig. 5, Table 1). For example, in Fig. 5B cleavage of TAME was tested, which is the substrate that has the same cleavage site as C4 and C2 and there was no cleavage inhibition by SALO, suggesting that SALO does not inhibit the catalytic activity of C1s and will very likely not affect the activity of C1s towards other substrates. We could not rule out, however, the possibility that SALO may interact with an exosite on C1s preventing therefore, the binding of full-length C4 and consequently its cleavage, all of which may not be detectable using TAME as a substrate. Thus, we measured the ability of rSALO to inhibit the cleavage of the natural C1s substrate, C4. Fig. 5C confirmed SALO has no effect on the catalytic activity of C1s on C4. Thus it is possible that SALO may act at the level of C1q, potentially displacing the serine proteases from the C1 complex, without inhibiting the binding of C1q to IgG or by inhibiting directly C1r activity. Further work is needed to determine the molecular mechanism of complement inhibition by SALO.

The implications and the potential impact of our findings, in addition to elucidating the role of SALO in sand fly feeding, are 1) SALO may be used as a therapeutic agent to prevent the pathogenic effects of complement; in particular in scenarios where the classical pathway plays an important initiating role in pathogenesis such as transplant acute hyper-rejection^30^, autoimmune hemolytic anemia^31^, and certain kidney diseases^32^; 2) SALO may protect the sand fly midgut from complement activity, previously demonstrated for triatomine and ticks^7^,^8^, and consequently may help *Leishmania* establishment and development inside the insect.

Given the essential role of the complement system in inflammatory processes and its modulatory involvement in many pathophysiological processes including acute and chronic inflammatory diseases, hematological and neurodegenerative disorders, cancer, ischemia/reperfusion (I/R) injuries, and sepsis^33^, the complement system is an attractive target for inhibitory drug development. Currently there are only two anti-complement drugs available on the market (recombinant C1 inhibitor and an anti-C5 monoclonal antibody), which are highly expensive, and considerable efforts are being made to identify additional candidate inhibitors for drug development^34^. Systemic inhibition of the complement system increases the risk of infections. Strategies that inhibit the complement system at early stages (e.g. at the level of the initiator complex C1) selectively block one or two pathways, leaving the other(s) intact. There are currently two classical pathway inhibitors (PIC1 and TNT003/009) that are in advanced stages of pre-clinical or early clinical development, respectively. PIC1 (Peptide inhibitor of complement C1) is a 15 amino acid peptide that specifically inhibits the initiation of the classical and lectin pathways of complement by binding to the collagen-like region of C1q and MBL, respectively, which blocks activation of the associated serine proteases (C1s/C1r or MASPs) and subsequent downstream complement activation^35^. TNT003, a murine anti-C1s monoclonal antibody, is a first-in-class classical pathway-specific inhibitor^36^. TNT009, the humanized version of the anti-C1s monoclonal antibody, is currently in a phase I clinical trial (ClinicalTrials.gov Identifier: NCT02502903). Additional classical pathway-specific therapeutic options are needed and will allow determination of relative affinity for targets and comparative effectiveness in each of many disease models, in order to have the best therapeutic option available to patients.

In an effort to contribute to the understanding of the biological function of this novel protein, we have identified the ability of SALO to specifically inhibit the human classical pathway of the complement system and determined partially the mechanisms involved. In comparison to natural anti-complement inhibitors including C1-INH which are large proteins^37^, SALO is a much smaller protein of only 11 kDa (with an apparent MW of 20 kDa in its active form). Unlike complement inhibitor calreticulin from parasites and ticks^38^ that shares significant activity and protein sequence homology to its human counterpart, the sequence of SALO is only present in sand flies of the genus *Lutzomyia*, and not in any other known organisms, including other sand fly genera and humans^27^. Another important aspect of SALO is that this protein alone could not elicit antibodies. Immunization of hamsters with SALO-coding DNA plasmids did not produce antibodies to the native protein^28^,^29^. In the current work we could only produce neutralizing antibodies in mice in the presence of a strong adjuvant. Therefore, as a therapeutic agent, rSALO may have an advantage due to its small size, potent specific anti-complement activity and low antigenicity.

The effects of sand fly inhibition of the complement system are not restricted to mammalian tissues. The anti-complement factors described in saliva of the kissing bug *Triatoma brasiliensis* protected the insect’s midgut from complement activity^7^. The removal of saliva containing the anti-complement activity resulted in severe damage to the insect’s midgut epithelium. In *Lu. longipalpis*, SALO may also protect the fly midgut and possibly protect the *Leishmania* parasite as well. Anti-SALO antibodies may remove SALO midgut protection therefore negatively affecting parasite establishment in the vector’s midgut. This could be envisioned as a potential transmission blocking vaccine where antibodies to a salivary protein may prevent the development of the parasite inside the fly gut and consequently prevent parasite transmission to another host. Further experiments are needed to establish if anti-SALO antibodies could represent an alternative strategy to prevent parasite transmission.

## Methods

### Ethics statement

All animal procedures were reviewed and approved by the National Institute of Allergy and Infectious Diseases (NIAID) Animal Care and Use Committee and handled in accordance to the Guide for the Care and Use of Laboratory Animals and with the NIH OACU ARAC guidelines and also approved under animal Protocol no. 87/2011 of Ethics Committee in Animal Experimentation (CETEA/UFMG).

### Reagents, purified proteins and antibodies

Complement protein C1s and C3 were purchased from CompTech. All proteins were stored at −75 °C. Human IgG was purchased from Sigma or purified from normal human serum as described before^39^.

Antibodies against complement proteins were purchased from Sigma (rabbit polyclonal anti-human C3c, rabbit polyclonal anti-human C4), CompTech (polyclonal anti-human C4, polyclonal anti-human C5, polyclonal anti-human C9) and Serotec (monoclonal anti-human C4b, anti-human C4-FITC conjugated IgG antibody). For ELISA and Western-blot assays, the secondary antibodies used were: phosphatase-conjugated donkey anti-mouse IgG (Chemicon), peroxidase-conjugated goat anti-mouse IgG (Sigma), peroxidase-conjugated goat anti-rabbit IgG (Sigma) and peroxidase-conjugated rabbit anti-goat IgG (Sigma).

### Sand fly salivary gland homogenate preparations

*Lu. longipalpis* sand flies were obtained from colonies at the Department of Parasitology, Federal University of Minas Gerais and from the Vector Molecular Biology Section, LMVR, NIAID, NIH. Insects were reared as described previously^40^. To obtain the SGH, salivary glands from 3- to 5-day-old unfed females were dissected in cold saline (0.9% NaCl) using a stereomicroscope and the extracted material was pooled and sonicated for 20 s. Alternatively, salivary glands were dissected from female *Lu. longipalpis* in PBS and frozen at −80 °C until use. Vials were thaw on ice, sonicated (4 × 20 pulses), and centrifuged at 10,000 × g for 3 minutes. Only supernatants were used in the experiments.

### Hemolytic Assays

To assess the effect of SGH or rSALO on complement activation, assays of classical pathway-mediated lysis were performed using antibody-coated sheep erythrocytes (EA). SGH or rSALO was mixed, on ice GHB^ + 2^ solution (5 mM HEPES, 145 mM NaCl, 0.15 mM CaCl_2_, 0.5 mM MgCl_2 _ and 0.1% gelatin, pH 7.4) or GVB^2 + ^(5 mM Veronal, 145 mM NaCl, 0.15 mM CaCl_2_, 0.5 mM MgCl_2_, 0.025% NaN3 and 0.1% gelatin, pH 7.3), respectively, with normal human sera (NHS) (final concentration 0.65%). EA (0.5 × 10^7 ^ cells) were added and the mix (62.5 μl total volume) was transferred to a 37 °C water bath and incubated for 30 min. To determine the extent of hemolysis, 250 μl of cold saline solution was added, the samples were centrifuged, and the optical density of the 200 μl supernatant was determined at 414 nm. The percent lysis was determined by subtracting the A_414 _in the absence of serum, and dividing by the maximum possible A_414_ determined by water lysis of the erythrocytes. The results were analyzed by ANOVA followed by Tukey test for statistics.

### Cloning and expression of recombinant sand fly salivary proteins in HEK 293-F cells

SALO and other sand fly salivary proteins were cloned into the VR2010-TOPO vector as described before^41^,^42^. Recombinant proteins were produced in Leidos Biomedical Research by transfecting HEK 293-F cells with DNA plasmids coding for the different sand fly salivary and incubated for 72 hours. The supernatant was purified as previously described using a HiTrap chelating HP column (GE Healthcare) charged with Ni_2_SO_4_^41^. Imidazole was removed from positive fractions by washing with PBS using a 10.000 MWCO Amicon filter (Millipore). Samples were further cleaned with a 100,000 MWCO amicon filter to remove any traces of surfactants and stored at −80 °C.

### Silver Staining

SDS-PAGE electrophoresis was performed with 4–12% NUPAGE gels (Life Technologies, Carlsbad, CA). Recombinant salivary proteins (100 ng each) mixed with two microliters of NUPAGE Sample Reducing Agent were heated up for 10 minutes at 70 °C and loaded onto the gel. See Blue Plus 2 Pre-stained Standard (6 μl; Life Technologies) was used as for molecular weight reference. Gel was silver stained using the SilverQuest™ Staining Kit (Life Technologies) and following the manufacturer’s recommendations.

## Multiple Sequence Alignment

The *Lu. longipalpis* SALO amino acid sequence was blasted against the NR database, matching to three other known salivary proteins: LJS169 and LJS192 from *Lu. longipalpis*^27^ and LayS36 from *Lu. ayacuchensis*^24^. Multiple sequence alignment was carried out with the ClustalW software.

### Molecular Sieving Chromatography of SGH and rSALO

Molecular sieving chromatography of salivary gland homogenates of *Lu. longipalpis* or rSALO was performed with a Superdex 75 column (3.2 mm × 30 cm) (GE Healthcare) running Tris-Buffered saline (150 mM NaCl, 20 mM Tris-Cl pH 7.4) pumped at 50 μl/min with a Spectra System P4000 pump (Thermo). Absorbance at 280 nm was measured with an Applied Biosystems UV detector model 785A, and fractions were collected at 1 min interval using a Probot x/y/z fraction collector from LCPacking.

### Antiserum against rSALO

Six to eight weeks old female Balb/c mice were injected three times every two weeks, intradermally in the ear with 5 μg of rSALO mixed (1:1 volume) with Magic™ Mouse Adjuvant (Creative Diagnostics, Shirley, NY) as recommended by the manufacturer. Another set of antibodies to rSALO used for immunoprecipitation studies was developed as follows: rSALO bands were excised from gel slabs (10% SDS-PAGE stained with coomassie blue), homogenized and subcutaneously inoculated in mice. Animals were immunized three times, every 15 days. Fifteen days after the last inoculation, blood was collected to obtain the rSALO antiserum.

### Blockage of SALO activity by rSALO antiserum

Hemolytic assays using SGH or rSALO in the presence of the rSALO antiserum were done. The assays were perfomed as described above, but before mixing the SGH or rSALO with the NHS in GVB^2 + ^, the inhibitors were incubated with 12.5 μl of different dilutions of the antiserum (1:10; 1:100; 1:1000 or 1:10000, in PBS). In the experiments using SGH and rSALO, 0.5 salivary gland pair and final concentration of 0.1 μM was used, respectively. A control was done mixing antiserum diluted 1:10 with the red blood cells, to check if it could affect the cells and cause hemolysis. The assay followed as described previously.

### Immuno-depletion of native SALO present in SGH

To remove the native form of SALO from SGH we employed an immunopreciptation approach using rSALO antiserum. Briefly, one ml of heat inactivated rSALO antiserum diluted in 20 mM phosphate buffer containing 1 M NaCl was applied in the top of a Protein-A sepharose resin (Sigma) column. After washing the column three times with the same buffer and three times with PBS, the resin containing IgG from mice serum was collected for use. A control resin was prepared in the same way, but using normal mouse serum instead of rSALO antiserum. Fifty μl of the treated resin were mixed with an equal volume of SGH in PBS containing the equivalent of 4 salivary lobes. The tubes were then incubated for 2 hours at room temperature to enable anti-rSALO to bind the native salivary protein. After centrifugation (1,700 × *g*, 1 min), 12.5 μl of the supernatant (equivalent to 0.5 salivary gland pair) were collected and used in hemolytic assays.

### ELISA assays for deposition of complement factors

ELISA microplates (COSTAR) were coated overnight in a moist chamber with 40 μg/ml purified human IgG diluted in carbonate/bicarbonate buffer (35 mM Na_2_CO_3_, 15 mM NaHCO_3_, pH 9.6). Wells were blocked in a two-step procedure, first by adding 200 μl of TNB solution (10 mM Tris, 140 mM NaCl and 3% BSA, pH 7.4) followed by a second block with 200 μl of TNBTC solution (10 mM Tris, 140 mM NaCl, 3% BSA, 0.05% Tween-20 and 5 mM CaCl_2_, pH 7.4), both during 30 min at room temperature. SGH (equivalent to 0-1 salivary gland pairs) or rSALO (0–0.45 μM) were then mixed in microcentrifuge tubes with NHS (final concentration 1%) diluted in HNCM solution (4 mM HEPES, 145 mM NaCl, 2 mM CaCl_2 _ and 1 mM MgCl_2_, pH 7.4). The mix (100 μl total volume) was transferred to IgG-coated wells and incubated for 30 min at 37 °C under constant agitation. In these conditions, serum incubation promotes classical pathway activation and consequent binding of complement factors over IgG-coated surface. After washing with washing buffer (10 mM Tris, 140 mM NaCl and 0.1% BSA, pH 7.4), wells were incubated with specific antibodies diluted in HN solution (10 mM HEPES and 140 mM NaCl, pH 7.4) for 30 min at room temperature under constant agitation. Dilutions of the antibodies were 1:2,500 for anti-C1q, anti-C5 and anti-C9 and 1:1,000 for anti-C3c and anti-C4. Wells were washed again and incubated for 30 min at room temperature under constant agitation with secondary peroxidase-conjugated antibody (anti-goat IgG peroxidase-conjugated antibody diluted 1:5,000 for C1q, C5b and C9 deposition or anti-rabbit IgG peroxidase-conjugated antibody diluted 1:2,500 for C3b and C4b deposition). After washing, plates were revealed by adding to each well 200 μl of developing buffer (50 mM sodium citrate, 50 mM Na_2_HPO_4_, 1 mg/ml OPD and 0.075% H_2_O_2_, pH 5.0). The absorbance at 450 nm was measure kinetically (every 30 sec) for 10 min at 37 °C in a microplate reader (Molecular Devices) and the rate of absorbance increase was calculated with SoftMax Pro 5.2 software. Values of mOD/min were used for analysis. The percent of each complement factor deposition was calculated by subtracting the rate of absorbance increase in the absence of serum, and dividing it by the rate of absorbance increase when no inhibitor was present. To evaluate the effects of SGH on pre-bound complement factors, a similar assay was carried. To allow the binding of complement proteins over the plate surface, wells were incubated with 1% NHS in HNCM solution without SGH. After washing, SGH (equivalent to 2 salivary lobes) alone was added to each well and the plate was incubated for 30 min at 37 °C. The plate was then washed and the deposition of C1q, C4b, C3b, C5b and C9 was evaluated as described before.

### Western blot assay to detect C4 cleavage

C4 cleavage due to complement activation was analyzed by Western blot. Hemolytic assays were conducted in the presence of SGH (equivalent to 2 salivary gland pairs), rSALO (0.72 μM) or no inhibitor. Ten μl of the supernatants were collected just after adding EA (0 min) and after 30 min of incubation at 37 °C and submitted to 10% SDS-PAGE. Western blot was carried using polyclonal anti-C4 (CompTech) diluted 1:2,000 as primary antibody and peroxidase-conjugated rabbit anti-goat IgG (Sigma) diluted 1:4,000 as secondary antibody. C4 hydrolysis could be observed by the appearing of C4b α’ chain.

### Proteolytic activity of C1s

The ability of rSALO (or rLJM17, control) to inhibit the proteolytic activity of C1s was assessed by a spectrophotometric assay for measuring the esterase activity of C1s using TAME as a serine protease substrate. Briefly, 0.6 μM C1s was mixed with 0.2 μM rSALO or rLJM17 in TAME reaction buffer (1.5 mM Nα-p-Tosyl-L-arginine methyl ester hydrochloride (Sigma), 150 mM NaCl, 1 mM EDTA, 50 mM Tris-HCl, pH7.6). C1s proteolytic activity was immediately assessed in the samples by determining A_247_ every minute for 10 min. Trypsin (0.2 μM) + TAME was used as a positive control and each LJM protein + TAME, without C1s, was included as negative controls.

The ability of rSALO (or rLJM17, control salivary protein) to inhibit the proteolytic activity of C1s was also assessed by incubating GVB++, C4 (0.86 μM), and C1s (0.0025−25 nM) in the presence or absence of SALO (1 μM), in a 10 μl total volume for 10 minutes at 37 °C. Sample buffer and reducing agent (Invitrogen) were added and C4 activation in the samples was assessed by SDS-PAGE.

### Serine protease inhibition assays

All assays were performed at 30 °C in triplicates. rSALO (120 nM) was pre-incubated with each enzyme to be tested for 5 minutes before the addition of the corresponding substrate. All enzymes used were of human origin, purified or recombinant. Thrombin and plasmin were purchased from Sigma (St. Louis, MO), Factor Xa from EMD Biosciences (La Jolla, CA), kallikrein from Fitzgerald Industries International (Concord, MA), uPA from Molecular Innovations (Southfield, MI) and sequencing grade trypsin from Roche (Chicago, IL). The amount of enzyme used in each assay is shown in the table. The assay buffers were: for trypsin and thrombin, 50 mM Tris pH 8, 150 mM NaCl, 20 mM CaCl_2_, 0.01% Triton X-100; for kallikrein and plasmin, 20 mM Tris pH 8.5, 150 mM NaCl, 0.02% triton X-100; for factor Xa, 20 mM Tris pH 8, 200 mM NaCl, 5 mM CaCl_2_, 0.1%BSA; for uPA, 20 mM Tris pH 8.5, 0.05% Triton X-100. The substrates used were Boc-Asp-Pro-Arg-AMC for thrombin and plasmin, Boc-Gln-Ala-Arg-AMC for trypsin and uPA (Sigma, St. Louis, MO) and methylsulfonyl-D-cyclohexylalanyl-Gly-Arg-AMC acetate for factor Xa and kallikrein (American Diagnostica Inc., Stamford, CT). All substrates were used in 250 μM final concentration for all the assays. Substrate hydrolysis rate was followed in a Spectramax Gemini XPS 96 well plate fluorescence reader (Molecular Devices, Sunnyvale, CA) using 365 nm excitation and 450 nm emission wavelength with a cutoff at 435 nm.

### Measurement of alternative pathway-mediated lysis of rabbit erythrocytes (E_R_) in the presence of rSALO

GVB, NHS (15% final), and 0.1 M MgEGTA (5 mM final concentration) in the presence or absence of 1 μM rSALO or LJM17 or 10 mM EDTA were mixed, on ice. E_R_ (1 × 10^6 ^cells), which are highly susceptible to the alternative pathway, were added and the mix (20 μl total) was transferred to a 37 °C water bath and incubated for 20 min. The percent lysis was determined by subtracting the A_414 _in the absence of serum, and dividing by the maximum possible A_414_ determined by water lysis of the erythrocytes.

## Radiolabeling of C3

C3 (50–100 μg; purchased from CompTech) were radiolabeled with 500 μCi of ^125^I for 30 min at 0 °C in a glass tube coated with Iodogen (Pierce Chemical Co, Rockford, IL). After incubation, the free ^125^I was removed by centrifugal desalting through a G25 column pre-equilibrated with GVB^43^. Specific activities for ^125^I-labeled C3 ranged from 3 to 4 μCi/μg.

### Measurement of alternative pathway-mediated C3b deposition in the presence of rSALO

Deposition of C3b on E_R_ was measured by mixing, on ice, GVB, C5-depleted serum (10% final), ^125^I-C3, 2.5 mM MgEGTA, and the rSALO or LJM17 protein being tested. The mixture was transferred to a 37 °C water bath for 15 min. The cells were pelleted rapidly (2 min, 10,000 × *g*) through 250 μl of 20% sucrose in GVBE in a microfuge tube to separate bound from free radiolabel. The bottoms of the tubes were cut off and the radioactivity in the cell pellet and the supernatant were measured to determine the percent ^125^I-C3b bound.

### Measurement of the lectin pathway of complement

Lectin pathway activity in normal human sera (in the presence or absence of rSALO or LJM17) was assessed using a Wieslab^R^ kit (Cat#MP320), following manufacturer’s instructions. Briefly, mannan-coated microtiter wells were incubated with an amount of NHS (1% NHS) capable of inducing ~50–60% of the maximum lectin pathway activity of the positive control kit sample, in the presence or absence of 0.5–2 μM rSALO or LJM17 for 1 hr at 37 °C. After washing, the wells were incubated with a phosphatase labeled antibody against C5b-9, followed by addition of substrate and reading OD (A405).

## Additional Information

**How to cite this article**: Ferreira, V. P. *et al*. SALO, a novel classical pathway complement inhibitor from saliva of the sand fly *Lutzomyia longipalpis*. *Sci. Rep*. **6**, 19300; doi: 10.1038/srep19300 (2016).

## Supplementary Material

Supplementary Information

## Figures and Tables

**Table 1:** Effect of rSALO (120 nM) on the enzymatic activity of various proteases.

Enzyme	Amount of enzyme used (nM)	Percent of enzyme activity in the presence of 120 nM of SALO	Percent of enzyme activity in control reaction
Thrombin	0.01	100.5 ± 2.9	103 ± 3.7
Factor Xa	0.12	101.5 ± 1.1	97.9 ± 1.2
Kallikrein	0.80	103.8 ± 2.6	108.9 ± 4.1
Trypsin	0.24	101.2 ± 1.1	96.9 ± 2.8
u-PA	0.70	93.7 ± 1.8	91.9 ± 1.1
Plasmin	0.50	96.6 ± 2.1	104 ± 2.7
