# Supplementary material for: SALO, a novel classical pathway complement inhibitor from saliva of the sand fly Lutzomyia longipalpis
Source: Sci Rep. 2016 Jan 13;6:19300. doi: 10.1038/srep19300 (PMC4725370; doi:10.1038/srep19300)
Supplement: Supplementary Information [file srep19300-s1.pdf]

Supplementary Material

SALO, a novel classical pathway complement inhibitor from saliva of the sand fly *Lutzomyia longipalpis*

Viviana P. Ferreira[1#], Vladimir Fazito Vale[2#], Michael K. Pangburn[3], Maha Abdeladhim[4], Antonio Ferreira Mendes-Sousa[2,4], Iliano V. Coutinho-Abreu[4], Manoochehr Rasouli[4], Elizabeth A. Brandt[4], Claudio Meneses[4], Kolyvan Ferreira Lima III[2], Ricardo Nascimento Araújo[2], Marcos Horácio Pereira[2], Michalis Kotsyfakis[5], Fabiano Oliveira[4], Shaden Kamhawi[4], Jose M. C. Ribeiro[6], Nelder F. Gontijo[2]*, Nicolas Collin[4,7]*, Jesus G. Valenzuela[4]*

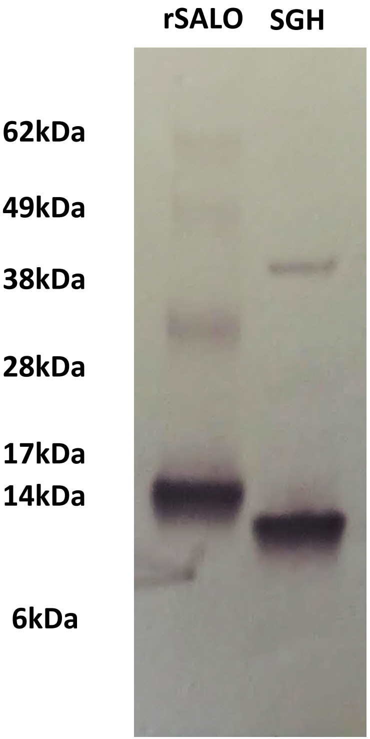
rSALO
SGH
62kDa
49kDa
38kDa
28kDa
17kDa
14kDa
6kDa

Supplemental Figure Legends

Supplementary Figure 1. Western blot analysis of recombinant SALO (rSALO) and *Lu. longipalpis* salivary gland homogenate (SGH). Proteins were run under reducing conditions on SDS-PAGE, transferred to a nitrocellulose membrane and reacted with rSALO antibodies generated in mice as described in detail in the methods section.
